# Impact of Sample Type on D-Dimer Screening

**DOI:** 10.21315/mjms2024.31.2.13

**Published:** 2024-04-23

**Authors:** Ellies Tunjung Sari Maulidyanti, Nur Vita Purwaningsih, Ainutajriani Ainutajriani, Rahma Widyastuti

**Affiliations:** Department of Medical Technology Laboratory, Faculty Health Science, University Muhammadiyah of Surabaya, Surabaya, Indonesia

**Keywords:** D-dimer, whole blood, plasma, fluorescent lateral flow immunoassay, clinical laboratory examinations

## Abstract

**Background:**

The quality of laboratory test results depends on various factors, including sample type selection. Blood samples, such as whole blood, plasma and serum are commonly used for most clinical laboratory examinations. D-dimer parameters are frequently analysed in haematology laboratories and serve as biomarkers for coagulation activation and fibrinolysis. This study aimed to assess the impact of using different sample types on the quality of D-dimer test results.

**Method:**

An observational analytical method was used. D-dimer examination was performed using the fluorescent lateral flow immunoassay method. The study sample consisted of 26 participants aged between 18 years old and 22 years old who had no blood disorders. Whole blood and ethylenediaminetetraacetic acid (EDTA) plasma samples were used for the examination of D-dimer levels.

**Results:**

D-dimer levels in 26 participants using whole blood samples had a mean value of 0.23 mg/L (230 ng/mL), while plasma samples yielded a mean value of 0.14 mg/L (140 ng/mL). D-dimer levels obtained from whole blood samples were higher than plasma samples but remained within the normal range of 0 mg/L–0.5 mg/L (0 ng/mL–500 ng/mL).

**Conclusion:**

The results showed that whole blood samples were more practical than plasma samples. Nevertheless, plasma samples gave results within the normal range of D-dimer values.

## Introduction

Laboratory tests play an important role in establishing an accurate diagnosis and prognosis for various diseases. To ensure the reliability of test results, laboratories must maintain a comprehensive approach across the three stages of examination: i) pre-analytical, ii) analytical and iii) post-analytical. The pre-analytical stage involves patient preparation, specimen identification, collection, processing, storage and delivery to the laboratory. The analytical stage includes equipment maintenance, calibration, performing the examination and monitoring accuracy. Finally, the post-analytical stage includes recording and reporting the results (1, 2).

Laboratory examinations are prone to errors that can significantly impact the accuracy of the results. Pre-analytical errors are the main source of diagnostic errors, accounting for up to 60%–70% of all errors, followed by the analytical stage (10%–15%) and post-analytical stage (15%–20%). Pre-analytical errors are often due to poor sample quality, incorrect patient identification, improper use of test tubes and specimen types that are not suitable in terms of laboratory parameters (3, 4).

Blood samples are the most common type used in clinical laboratory testing and include whole blood, plasma and serum (5). Whole blood consists of the liquid parts of blood: red blood cells (RBCs), white blood cells (WBCs), platelets and other plasma cell elements that can cause clotting under certain circumstances (6). Plasma is the cell-free fluid part of blood that contains clotting factors, while plasma refers to the cell-free fluid without clotting factors (7).

Decru et al. (8) demonstrated that sample type, specifically whole blood and plasma, can significantly affect the sensitivity of SARS-CoV-2 IgM screening using lateral flow assays (LFA) rapid tests. Whole blood samples showed lower sensitivity compared to plasma samples, with rates of 87.9% and 90.9%, respectively. Similarly, Kesuma et al. (9) reported that whole blood, plasma and serum samples showed significant differences in blood glucose levels, with ethylenediaminetetraacetic acid (EDTA) plasma samples showing higher glucose levels compared to whole blood and serum samples. Thus, these studies highlight the importance of considering the appropriate type of blood sample for specific laboratory parameters.

One of the laboratory parameters using blood samples is D-dimer, which is frequently examined in haematology laboratories and serves as a biomarker for coagulation activation and fibrinolysis. D-dimer is a product of fibrin degeneration and is used to identify blood clot formation/thrombotic abnormalities and to assess the fibrinolytic process (10, 11). Elevated D-dimer levels indicate the presence of a thrombus but do not indicate the location of the abnormality, ruling out various causes (12, 13). Variables that may affect D-dimer results include anticoagulant therapy (which can lead to false negative findings), age, pregnancy (increases D-dimer levels) and samples subjected to haemolysis caused by clotting or inappropriate handling (which leads to false positive findings) (14, 15).

In the context of COVID-19, D-dimer is used as a reliable biomarker to predict mortality in COVID-19 patients (16). Moreover, elevated D-dimer levels in severe COVID-19 infection may be attributed to an indirect response to the cytokine inflammatory reaction, resulting in an imbalance between coagulation and fibrinolysis in the alveoli and subsequently activating the fibrinolysis system (14, 15). This study aimed to determine the effect of using different sample types on the quality of D-dimer test results.

## Methods

### Research Design

This study used an observational analytical method to examine D-dimer levels. Fluorescent lateral flow immunoassay (FIA) method was used for D-dimer examination.

### Place and Time of Research

This study used an observational analytical method to examine D-dimer levels. FIA method was used for D-dimer examination.

### Research Tools and Materials

The following tools were used in this study: FIA method, centrifuge, 3 cc syringe, vacutainer tube with 3.2% Na citrate and tourniquet.

The study material consisted of a sample of 26 participants aged between 18 years old and 22 years old and free from blood disorders. The Slovin formula was used to determine the sample size. The examination samples included whole blood and citrated plasma for D-dimer level examination.

### Test Procedure

Tests shall be conducted at room temperature (−25 °C) as follows:

Step 1: Preparation - insert the a Secure Digital card into the equipment.
Step 2: Sample collection - collect 50 μL of plasma or 80 μL of whole blood using a transfer pipette and add it into a buffer tube.
Step 3: Mix the specimen thoroughly by tapping or inverting the buffer tube.
Step 4: Take 80 μL of the sample mixture and insert into the well of the test cartridge.
Note: Steps 2–4 must be completed within 1 min to ensure the accuracy of the test results.
Step 5: Incubate the cartridge for 3 min then read on the device.

### Data Analysis

Data were analysed using an independent *t*-test using SPSS version 23.0 software.

## Results

### General Characteristics of Research Subjects

The research sample comprised 26 participants aged 18 years old–22 years old who had no blood disorders.

### Comparison of Results Using Whole Blood and Plasma Specimens

Table 1 presents the D-dimer examination findings, which were processed using SPSS software through an independent *t*-test. At first, the normality of the data was assessed using the Shapiro-Wilk test, with the result of plasma data being 0.00 and whole blood data being 0.002. Next, the data were analysed using an independent *t*-test, which showed a significant *P*-value of 0.000. D-dimer levels obtained from whole blood specimens were higher compared to plasma specimens. The standardised coefficient for whole blood was 95% CI: 1.98, 2.62 (*P* = 0.000), while for plasma it was 95% CI: 1.22, 1.69 (*P* = 0.000).

## Discussion

Examination of D-dimer levels in 26 participants showed that the mean D-dimer value was 0.23 mg/L (230 ng/mL) for whole blood samples and 0.14 mg/L (140 ng/mL) for plasma samples. The D-dimer levels obtained using whole blood samples were higher compared to plasma samples. Both types of samples were still within the normal range of D-dimer values, which is 0 mg/L −0.5 mg/L (0 ng/mL–500 ng/mL). According to the recommendations of the WHO, plasma samples can be used for D-dimer and several other examination parameters without affecting the accuracy of the results, such as alanine aminotransferase (ALT), albumin, bilirubin and cholesterol. In this study, the FIA method was used, which accommodates plasma and whole blood specimens (14, 16). However, the results obtained from whole blood samples were higher compared to plasma samples, possibly due to the presence of fibrinogen in plasma, which may affect D-dimer levels. Previous studies (14, 17, 18) have indicated that the use of plasma samples could be less sensitive for D-dimer analysis due to the presence of fibrinogen.

Whole blood, which comprises 6%–8% of body weight, is made up of both liquid and solid components (19). The liquid component, also known as plasma, accounts for about 55% of the total blood volume and consists of water and various chemical compounds. Meanwhile, the solid component, which accounts for about 45% of the total blood volume, consists of blood cells, including erythrocytes, leucocytes and platelets (20). Whole blood is widely used as a sample for several laboratory examination parameters and serves as the gold standard for point of care testing. Similarly, Kesuma et al. (9) reported that EDTA serum and plasma samples can increase glucose levels compared to whole blood samples.

Blood plasma is a clear yellowish liquid containing 92% water, a complex mixture of organic and inorganic substances (21). In addition, plasma also contains nutrients, blood gases, electrolytes, minerals, hormones, vitamins and waste substances (22). Plasma is obtained by separating blood cells from whole blood through centrifugation. The composition of clotting factors in plasma may vary depending on the type of anticoagulant used. Anticoagulants prevent the formation of blood clots and the conversion of fibrinogen into fibrin (23).

D-dimer testing is commonly used for the diagnosis of deep vein thrombosis, pulmonary embolism and disseminated intravascular coagulation (DIC) (24). D-dimer is a by-product of the clotting and blood breakdown process in the blood. It is released when a blood clot begins to disintegrate and platelets bind to the D subunit, forming a bond (25). Through D-dimer and other factors, many platelets aggregate and form bonds with each other, such as clots formed from fibrin. As the body heals, the clot begins to break down, leading to the release of D-dimer from platelets (26).

## Conclusion

This study shows that using whole blood specimens is more practical than plasma samples for D-dimer examination. Nevertheless, the D-dimer levels obtained from plasma specimens were still within the range of normal D-dimer values. Despite the various advantages of this study, it is important to recognise its limitations, which only focus on the comparison of specimen types. Future studies should explore additional factors that influence D-dimer results and investigate the effectiveness of different sample types in D-dimer screening.

## Figures and Tables

**Figure 1:**
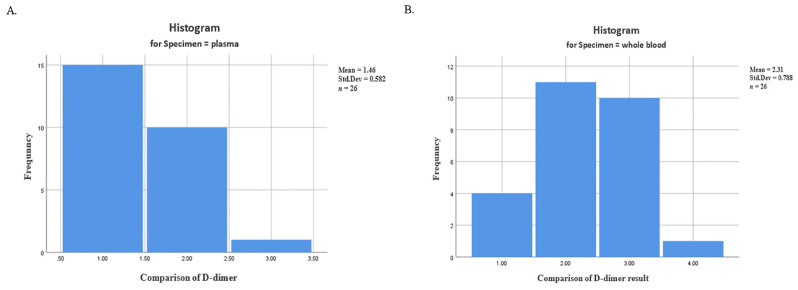
The difference in D-dimer histogram results. A. Whole blood specimen, B. Plasma specimen

**Table 1:** Comparison of D-dimer results using whole blood and plasma specimens

Variable	Standard error	Sig	95% CI	

			Lower bound	Upper bound
Whole blood	0.15	0.000	1.98	2.62
Plasma	0.11	0.000	1.22	1.69

Notes: Dependent variable: D-dimer, whole blood and plasma; significant at *P* < 0.05
